# The brief data of the relation of living as commuters and quality of life

**DOI:** 10.1016/j.dib.2019.104540

**Published:** 2019-09-25

**Authors:** Muhammad Fitri Rahmadana, Gaffar Hafiz Sagala

**Affiliations:** Faculty of Economics, Universitas Negeri Medan, Indonesia

**Keywords:** Commuters, Quality of life, Demography, Preference

## Abstract

The dataset presented here is useful for identifying the relation of living as commuters and the quality of life. This dataset separates commuters into several groups according to their gender, marital status, educational background, and occupation to enrich the demography data and to give more insight. Data was collected from commuters who work in Medan, North Sumatra, Indonesia. Questionnaire-based survey with proportional random sampling has collected from 384 respondents by accidental sampling. We use statistic descriptive, median-test, Kruskal-Wallis, and spearman-rank correlation to analyze the data. The data shows there is any different response regarding the quality of life between gender, marital status, educational background, and occupation among commuters. The researchers found at least three dominant factors which make the differences, that is the time limitation for social interaction in the neighborhood, time limitation regarding quality interaction as parents, and time limitation for themselves to have quality time. However, the commuters have several reasons why they still survive in the situation. From ten factors proposed, there are only three factors which have significant relation with three dominant factors of commuters life problem which suggested previously. That is 1) Time Compliance with Income; 2) Income per Month, and 3) Fairness Travel Time to Work Location.

**Table:** Specifications Table

Subject	*Economics and Econometrics/Geography, Planning and Development.*
Specific subject area	*Demography of Commuters, Commuters Life, Quality of Life.*
Type of data	*Table*
How data were acquired	*The survey conducted using questionnaire* (Appendix 1*). The instrument contains descriptive data of respondents and 5-Likert scale questionnaire regarding quality of life. The data was distributed in*Appendix 2.
Data format	*Raw Data, Descriptive Analysis and Analyzed Statistical Data.*
Parameters for data collection	*The sample collected from the population who live as commuters in Medan, North Sumatera, Indonesia. Three hundred eighty-four respondents have collected from 30-entrance way to Medan City from each region around. Furthermore, the collected data were tabulated based on gender, marital status, educational background, and occupation to analyze the contrast of phenomenon of quality of life among commuters.*
Description of data collection	*Data was collected using 5-Likert scale questionnaires for quality of life (see*Appendix 3*for Validity and Reliability), commuters factors with various scale and individual characteristics with accidental sampling method and proportional random sampling technique. (*Appendix 4*)*
Data source location	*Medan, North Sumatra, Indonesia*
Data accessibility	*Data which contained in this article are accesible in Mandeley Data:*https://doi.org/10.17632/df6vx9nzzm.3

**Table:** 

**Value of the Data** • The data in this article displays a brief exploration regarding commuters preferences toward their quality of life. • The dataset describes the gap of preferences between a sample which grouped in gender, marital status, educational background, and occupation. • The data provide the fruitful result because the data figured heterogeneous demography background of the sample. • The data is valuable for further research regarding quality of life, index of happiness, productivity, and welfare in the region or even as proxies of another area which have similar characteristic. • For researchers who interested in public sector development, we present a dataset that is valuable for predicting how commuters define their quality of life and the reason for its definitions. • The dataset can be further analyzed in the future using an advanced method of data analysis or even new data format. • The figure of data is valuable for the reason of decision-making regarding public transportation investment, traffic regulation, and income regulation to control the index of happiness based on the quality of life.

## 1 Data

The data proposed here resulted from surveying commuters who were working in Medan City while they live in another city around Medan. The previous research has shown that commuters life has several impacts on quality of life [1], [2], [3], [4], and demography of the commuters also contribute to resulting the differences of commuters' perspective regarding their quality of life [1], [3], [5], [6]. Therefore, we identify the perception of commuters regarding their quality of life and why they choose those circumstances. Then we separated the data refers to their valuable characteristics, such as gender, marital status, educational background, and occupation for better display of data. Table 1 presents the figure of quality of life regarding the demography of respondents. The author uses the median test and Kruskal-Wallis to identify the difference of responses of each group. It contains the signification number of differences in commuter perception regarding their quality of life according to gender, marital status, educational background, and occupation. The detail of the respondent's demography and its figure of responses are distributed in Table 2, which probable the readers to analyze what factors in demographics which may affect the quality of life among commuters. Furthermore, for advanced analysis, the author used rank-spearman correlation to identified the relationship between quality of life and the antecedents (Table 3). From Table 3, we can indicate there are four aspects which influencing sample to justify their quality of life, that is: work time compliance with income, income per month, ease of transportation mode, fairness travel time to work location.

**Table 1:** Figure of commuters perception regarding quality of life.

Quality of Life	Commuters							
	Gender		Marital Status		Educational Background		Occupation	
	Z	Sig	Chi-Square	Sig	Chi-Square	Sig	Chi-Square	Sig
Your life will be much better if you work not become a commuters	−4.507	0.000**	2.092	0.553	2.827	0.727	2.448	0.654
You don't have enough time to do social interaction in your neighborhood	−2.976	0.003**	18.085	0.000**	15.07	0.010**	31.926	0.000**
Your quality as a parent is disturbed because you do not have enough time to interact with your children	−2.058	0.040*	11.797	0.008**	15.838	0.007**	31.696	0.000**
You don't have time to think about yourself	−0.147	0.883	8.534	0.036*	15.612	0.008**	27.04	0.000**
Your family life is disrupted because you do not have enough time to interact with your partner so that many things cannot be discussed because of this limited time	−2.053	0.040*	6.687	0.083	9.770	0.082	24.178	0.000**
Your overall life satisfaction as a commuters is reduced because you no longer have much time to channel hobbies and other activities besides routine activities.	−1.334	0.182	4.013	0.260	4.466	0.484	10.204	0.037*
You feel that you have individuals stress with activities as a commuters	−1.691	0.091	2.491	0.477	5.009	0.415	15.341	0.004**
Even though your work is within reasonable limits but the time you allocate to travel as a commuters makes you feel overworked	−0.162	0.872	4.884	0.180	4.533	0.475	9.863	0.043*

Notes: ** Significant at the 0.01 level; * Significant at the 0.05 level.

**Table 2:** Statistic descriptive.

	Gender	N	Mean	SD	Marital Status	N	Mean	SD	Educational Background	N	Mean	SD	Occupation	N	Mean	SD
Your life will be much better if you work not become a commuters	Male	285	3.410	0.776	Married	289	3.498	0.804	Primary School	13	3.230	0.832	Civil Servant	59	3.491	0.817
	Female	99	3.778	0.693	Single	81	3.494	0.615	Junior High School	44	3.546	0.697	Police/Military	15	3.733	0.799
					Widower	3	4	0	Senior High School	229	3.507	0.776	Private Employees	110	3.518	0.798
					Widow	11	3.636	1.027	Diploma	24	3.541	0.779	Self Employees	67	3.433	0.783
									Under Graduate	72	3.5	0.805	Others	133	3.511	0.724
									Post Graduate	2	4	0				
You don't have enough time to do social interaction in your neighborhood	Male	285	3.154	0.772	Married	289	3.135	0.815	Primary School	13	2.923	0.862	Civil Servant	59	2.915	0.749
	Female	99	3.444	0.836	Single	81	3.568	0.669	Junior High School	44	3.046	0.569	Police/Military	15	2.867	0.915
					Widower	3	3.333	0.577	Senior High School	229	3.328	0.801	Private Employees	110	3.418	0.806
					Widow	11	3.182	0.603	Diploma	24	3.25	0.944	Self Employees	67	2.925	0.804
									Under Graduate	72	3.056	0.803	Others	133	3.406	0.697
									Post Graduate	2	4	0				
Your quality as a parent is disturbed because you do not have enough time to interact with your children	Male	285	3.147	0.879	Married	289	3.128	0.939	Primary School	13	2.846	1.068	Civil Servant	59	2.966	0.909
	Female	99	3.364	0.963	Single	81	3.482	0.743	Junior High School	44	3.136	0.509	Police/Military	15	3.066	0.704
					Widower	3	3.667	1.155	Senior High School	229	3.301	0.951	Private Employees	110	3.309	0.993
					Widow	11	3	0.632	Diploma	24	3.292	0.999	Self Employees	67	2.761	0.889
									Under Graduate	72	2.944	0.837	Others	133	3.459	0.744
									Post Graduate	2	4	0				
You don't have time to think about yourself	Male	285	3.221	0.890	Married	289	3.173	0.904	Primary School	13	2.615	0.506	Civil Servant	59	2.966	0.870
	Female	99	3.232	0.818	Single	81	3.432	0.757	Junior High School	44	3.273	0.585	Police/Military	15	3.2	1.014
					Widower	3	3.667	1.155	Senior High School	229	3.323	0.927	Private Employees	110	3.390	0.929
					Widow	11	2.909	0.302	Diploma	24	3.25	1.113	Self Employees	67	2.865	0.736
									Under Graduate	72	2.986	0.721	Others	133	3.383	0.795
									Post Graduate	2	3	0				
Your family life is disrupted because you do not have enough time to interact with your partner so that many things cannot be discussed because of this limited time	Male	285	3.137	0.899	Married	289	3.132	0.926	Primary School	13	2.769	0.599	Civil Servant	59	3.016	0.881
	Female	99	3.343	0.835	Single	81	3.407	0.755	Junior High School	44	3.046	0.776	Police/Military	15	2.933	0.884
					Widower	3	3.667	1.155	Senior High School	229	3.266	0.895	Private Employees	110	3.309	0.864
					Widow	11	3	0	Diploma	24	3.333	0.868	Self Employees	67	2.806	1.019
									Under Graduate	72	3.069	0.954	Others	133	3.391	0.757
									Post Graduate	2	3	0				
Your overall life satisfaction as a commuters is reduced because you no longer have much time to channel hobbies and other activities besides routine activities.	Male	285	3.232	0.762	Married	289	3.239	0.805	Primary School	13	3.231	0.599	Civil Servant	59	3	0.643
	Female	99	3.394	0.831	Single	81	3.419	0.722	Junior High School	44	3.159	0.776	Police/Military	15	3.333	0.488
					Widower	3	3.333	0.577	Senior High School	229	3.341	0.793	Private Employees	110	3.327	0.879
					Widow	11	3.091	0.539	Diploma	24	3.167	1.007	Self Employees	67	3.239	0.923
									Under Graduate	72	3.181	0.699	Others	133	3.361	0.678
									Post Graduate	2	3	0				
You feel that you have individuals stress with activities as a commuters	Male	285	3.284	0.843	Married	289	3.291	0.881	Primary School	13	3.385	0.650	Civil Servant	59	2.966	0.889
	Female	99	3.464	0.825	Single	81	3.469	0.709	Junior High School	44	3.25	0.839	Police/Military	15	3.067	1.099
					Widower	3	3.667	1.154	Senior High School	229	3.389	0.839	Private Employees	110	3.436	0.873
					Widow	11	3.273	0.467	Diploma	24	3.333	0.817	Self Employees	67	3.358	0.811
									Under Graduate	72	3.194	0.898	Others	133	3.421	0.730
									Post Graduate	2	3	0				
Even though your work is within reasonable limits but the time you allocate to travel as a commuters makes you feel overworked	Male	285	3.356	0.855	Married	289	3.349	0.877	Primary School	13	3.077	1.115	Civil Servant	59	3.068	0.828
	Female	99	3.404	0.727	Single	81	3.457	0.633	Junior High School	44	3.341	0.888	Police/Military	15	3.533	0.834
					Widower	3	4	0	Senior High School	229	3.402	0.840	Private Employees	110	3.464	0.809
					Widow	11	3.091	0.539	Diploma	24	3.333	0.761	Self Employees	67	3.358	0.949
									Under Graduate	72	3.333	0.692	Others	133	3.414	0.739
									Post Graduate	2	4	0

**Table 3:** Quality of life among commuters and its antecedents.

	Affordability of Housing Costs	House Eligibility	Dependency Ratio	Income as a Commuters is More Bigger	Income as a commuters is Worthy	Work Time Compliance with Income	Income per Month (USD)	Ease of Transportation Mode	Comfort Travel to Work Locations	Fairness Travel Time to Work Location
Your life will be much better if you work not become a commuters	0.000^a^	0.511	0.908	0.000^a^	0.037^b^	0.258	0.359	0.001^a^	0.732	0.286
You don't have enough time to do social interaction in your neighborhood	0.332	0.323	0.764	0.141	0.777	0.079	0.000^a^	0.025^b^	0.407	0.035^b^
Your quality as a parent is disturbed because you do not have enough time to interact with your children	0.289	0.409	0.339	0.691	0.67	0.004^a^	0.000^a^	0.113	0.859	0.046^b^
You don't have time to think about yourself	0.008^a^	0.628	0.629	0.464	0.287	0.012^b^	0.006^a^	0.102	0.670	0.000^a^
Your family life is disrupted because you do not have enough time to interact with your partner so that many things cannot be discussed because of this limited time	0.212	0.260	0.174	0.925	0.647	0.014^b^	0.000^a^	0.016^b^	0.057	0.053
Your overall life satisfaction as a commuters is reduced because you no longer have much time to channel hobbies and other activities besides routine activities.	0.203	0.304	0.953	0.201	0.097	0.075	0.752	0.077	0.129	0.090
You feel that you have individuals stress with activities as a commuters	0.447	0.330	0.217	0.742	0.100	0.051	0.332	0.296	0.265	0.901
Even though your work is within reasonable limits but the time you allocate to travel as a commuters makes you feel overworked	0.165	0.292	0.550	0.630	0.925	0.002^a^	0.037^b^	0.108	0.521	0.036^a^

a Correlation is significant at the 0.01 level (2-tailed).

b Correlation is significant at the 0.05 level (2-tailed).

## 2 Experimental design, materials and methods

The commuters in this data are someone who has work location in Medan City while they live in another city around Medan. Medan is the capital city of North Sumatera and the 4th largest city in Indonesia, which has growing industrial clusters. People who work in Medan, mostly, come from another region around Medan because of the high price of the property in Medan. Therefore, to do their job, they need to take daily commuting at least 60–90 minutes to arrive at their workplace. This data distributed the commuters into several groups based on their demography, such as gender, marital status, educational background, and occupation. Researchers try to provide valuable data refer to the explorable value that already exists on the characteristic of the sample. Moreover, we analyze the data from each factor of variables to gain the detail variation of the relationship between variables.

The data was collected using 5-Likert-scale questionnaire. The questionnaire was distributed to the enumerator who spread into 30-entrance way to Medan City from each region around. Enumerator uses proportional random sampling to collect the data and accidental sampling to selecting the sample [7]. From those methods, researchers have obtained 384, which ready to be analyzed. After the dataset was collected, it was tabulated by the primary statistic method, mean and standard deviation, to analyze the descriptive statistics. From the figure of the statistic descriptive, we can predict the pattern and tendencies of data based on the demography of the sample. The descriptive data is observable in Table 2.

Furthermore, researchers use a median test to analyze commuters' quality of life according to gender and Kruskal Wallis to analyze commuters' quality of life regarding marital status, educational background, and occupation [8]. In this stage, we exploring the differences of responses among commuters refers to their characteristics by observing z-score and chi-square value to justify the significance of differences (Table 1). In the final stage, we use Spearman's Rank correlation to produce a correlation matrix between commuters' quality of life with its determinant factors, which can be observed in Table 3. Researchers use the methods to identify the relation between variable from each factor from both variables. The correlation data will show a detailed figure of the relationship, which is fruitful for interpretation. To support the accuracy of data analysis and practicality, we use IBM SPSS 22 to examine the dataset [9].

## Footnotes

Appendix A Supplementary data to this article can be found online at https://doi.org/10.1016/j.dib.2019.104540.
